# A novel thermostable lytic phage vB_EF_Enf3_CCASU-2024-3 against clinical *Enterococcus faecium* and *Enterococcus faecalis*

**DOI:** 10.1186/s13568-025-01871-z

**Published:** 2025-04-26

**Authors:** Rana M. Amr, Amr S. Bishr, Bishoy T. Saad, Mohammad Y. Alshahrani, Khaled M. Aboshanab, Nadia A. Hassouna

**Affiliations:** 1 Department of Microbiology and Immunology, Faculty of Pharmacy, Ain Shams University, Cairo, 11566 Egypt; 2 Department of Bioinformatics, HITS Solutions Co, Cairo, 11765 Egypt; 3 Central Labs, King Khalid University, P.O. Box 960, AlQura’a, Abha, Saudi Arabia; 4 Department of Clinical Laboratory Sciences, College of Applied Medical Sciences, King Khalid University, P.O. Box 61413, 9088 Abha, Saudi Arabia

**Keywords:** Virulence factor, Antibiotic resistance, Bacteriophage, *Enterococcus faecium*, *Enterococcus faecalis*, Stability

## Abstract

Enterococci, Gram-positive bacteria, have become a major concern in healthcare settings due to their significant virulence and antibiotic resistance. This research focuses on isolating, phenotypic, and genotypic analysis of enterococci-specific lytic phages to be used as potential candidates in combating multidrug-resistant (MDR) *Enterococcus* clinical isolates. The virulence of Enterococcus isolates was analyzed by testing for gelatinase and biofilm formation. The phage(s) was isolated from a sewage sample, then purified, propagated, and physiochemically analyzed. The phage was examined using transmission electron microscopy, and the whole genome sequence (WGS) was performed. Sixety-five clinical enterococci including, 27 (41.5%), 33 (50.7%) 3 (4.6%), and 2 (3%) *E. faecalis*, *E. faecium*, *E. avium*, and *E. durans*, respectively were isolated. Linezolid, teicoplanin, chloramphenicol, and vancomycin exhibited the lowest resistance. Twenty-five (38.5%) isolates were both gelatinase- and biofilm-producers. A novel lytic vB_EF_Enf3 phage belonging to *Caudoviricetes* class, characterized by an icosahedral head with a diameter of 100 ± 5 nm and a tail measuring 70 ± 5 nm in length was isolated. The phage demonstrated good thermal stability, and viability across various pH levels and exhibited a broad- spectrum of activity against *E. faecium* and *E. faecalis*. The vB_EF_Enf3 phage (36,202 bp length) harbored 36 open reading frames (ORFs) with a GC content of 34.4% (GenBank accession, PP747318). In conclusion, a novel thermostable lytic bacteriophage vB_EF_Enf3, belonging to class *Caudoviricetes,* was isolated from sewage showing broad-spectrum potent lytic activity against *E. faecium* and *E. faecalis* and maintained stability under various extreme conditions, including temperature, and pH fluctuations.

**Supplementary Information:**

The online version contains supplementary material available at 10.1186/s13568-025-01871-z.

## Introduction

Reports published in past years point to enterococci as an important cause of acquiring nosocomial infection (Hegstad et al. 2014). Despite being commensal bacteria that normally inhabit the human digestive system as part of the normal flora, these Gram-positive bacteria have acquired resistance genes through transposons and horizontal gene transfer (Vergis et al. 2002). Consequently, they have developed resistance to many antibiotics and have become the third most common origin of nosocomial infections (Shokoohizadeh et al. 2018). To survive the threat of being expelled by bowel movements, these intestinal bacteria possess various virulence factors that allow them to adhere to host tissues and persist (Jett et al. 1994). These factors, including enterococcal surface protein (ESP), cytolysin (CylA), and gelatinase (GelE), play critical roles in tissue invasion, colonization, and biofilm formation. These mechanisms significantly contribute to disease development by enabling the bacteria to evade the immune system and tolerate antimicrobial agents (Saffari et al. 2017; Shahi et al. 2020). These bacteria have become the origin of diseases such as urinary tract infections (UTIs), endocarditis, and bacteremia (Yang et al. 2020; EVREA-Phage 2024). Numerous infections, such as endocarditis, urinary tract infections, prostatitis, intra-abdominal infections, as well as nosocomial infections, are brought on by *Enterococcus faecalis* and *E. faecium* (Holmberg and Rasmussen 2016). They also produce concomitant bacteremia and become resistant to most of the antimicrobial agents used in their treatment. In addition, they form a biofilm on various implanted devices, as well as produce a variety of virulence factors, rendering their management challenging (Fiore et al. 2019; Lin et al. 2023). The incidence of *E. faecium* has significantly increased in recent years, particularly in resistant strains of enterococci, even though *E. faecalis* is often more common (Saba Copur et al. 2016). The two *Enterococcus* species most seen in human infections are *E. faecalis* and *E. faecium*. In addition, the emergence of vancomycin-resistant *E. faecalis* and *E. faecium* is globally increasing, particularly in hospital-acquired settings, imposing a medical hazard with limited therapeutic options (Lin et al. 2023).

The widespread bacterial resistance arising from the excessive use of antibiotics results in the growing demand for alternative treatments (Jett et al. 1994). This resistance has culminated in the development of very robust bacteria that are difficult to eradicate using conventional antibiotics (Jett et al. 1994). Our objective is to find novel approaches to tackle this issue. In 1915, a promising alternative began to emerge: bacteriophages. These biological entities offer a novel approach to combating resistant bacteria safely without harming human health. One of the key advantages of bacteriophages is their specificity; they target and attack only their specific bacterial hosts, providing a precise and effective means of bacterial eradication (Yang et al. 2020; EVREA-Phage 2024). Bacteriophages have become widely used in the treatment of many diseases, such as chronic venous leg ulcers caused by *P. aeruginosa* and *E. coli* and the FDA has clarified that the use of the phage cocktail has no negative effects (Rhoads et al. 2009). It has begun to be used in the treatment of chronic otitis externa caused by *P. aeruginosa* infection, and this was proven by phase II clinical trials in Europe (Al-Zubidi et al. 2019). It is important to note that bacteriophages are classified into two types: lytic and lysogenic. Lytic phages are typically utilized in treatments due to their ability to destroy bacterial cells. On the other hand, lysogenic phages are generally not used in treatment. However, advancements in genetic engineering have made it possible to convert lysogenic phages into lytic ones, thereby expanding their potential for therapeutic applications (Mazaheri Nezhad Fard et al. 2010; Bolocan et al. 2019).

Bacteriophages replicate within bacterial cells by commandeering the host's cellular machinery to generate progeny.to maximize the use of host resources, many phages encode auxiliary proteins that do not directly contribute to genome replication or particle assembly but instead modify the bacterial physiology to enhance phage replication. *Enterococcus faecalis* contains a low-complexity transposon that harbors the essential genes required for phage adsorption, as well as the replication and transcription of phage DNA (Chatterjee et al. 2020). Accordingly, this study aimed to isolate promising lytic phage(s) that target clinical isolates of enterococci. Given the widespread presence of enterococci and the nature of bacteriophages to coexist with their bacterial hosts, we have chosen to isolate this phage from hospital sewage water, where enterococci are commonly found. This approach leverages the natural association between bacteriophages and their host bacteria, facilitating the identification of effective lytic phages to be considered for potential clinical use against *Enterococcus* infections in humans.

## Materials and methods

### Collection of bacterial isolates

A total of 102 potential clinical *Enterococcus* isolates, collected from patients for routine checkups in Al-Demerdash and Al Kasr Al-Einy hospitals, were included in the study. The isolates inoculated in brain heart infusion (BHI) broth for propagation and isolated on BHI agar plates the colonies appear like small white colonies on the surface of the plate (Werner et al. 2019).

### Identification of the collected isolates:

A microscopic examination was conducted using Gram staining. Examination of *Enterococcus* colonies show Gram-positive cocci arranged singly, in pairs, or in short chains (Saba Copur et al. 2016; Dreyer et al. 2024). Colonies were identified as enterococci by being catalase-negative and growth in 6.5% NaCl (Dreyer et al. 2024). In the Bile-Esculin Azide test, *Enterococcus* colonies give pinpoint brownish colonies surrounded with a black halo (Suwantarat et al. 2014). Species-level identification was conducted using VITEK-2C (bioMérieux, France) at El Demerdash Hospital in Cairo, Egypt, following the manufacturer’s instructions. Calibration was carried out utilizing the standard strain *Escherichia coli* ATCC 25922 (Salah et al. 2021; Kim et al. 2023).

### Antibiotic susceptibility test

The collected isolates were tested using Kirby Bauer disk diffusion method and Muller Hinton agar medium by making a suspension equivalent to the half McFarland for susceptibility to disc including, vancomycin (30 μg), teicoplanin (30 μg), erythromycin (15 μg), ampicillin/ sulbactam (10 μg), linezolid (30 μg), ciprofloxacin (5 μg), chloramphenicol (30 μg), and doxycycline (30 μg). The vancomycin (VAN) susceptibility test was confirmed by measuring the MIC using the micro broth dilution method and *S. aureus* ATCC®29213 and *E. faecalis* ATCC 29212 were employed for quality control according to standard guidelines (Hegstad et al. 2014; CLSI 2021).

### Phenotypic detection of virulence markers of enterococci

#### Determination of gelatinase production

Pure colonies of enterococci were inoculated in a 5 mL tube of nutrient broth with 3% gelatin and then incubated at 37 °C overnight. After incubation, the tubes were refrigerated at 4 °C for half an hour. Positive tubes appeared liquefied. On the other hand, negative tubes appeared solidified (Fahmy et al. 2021).

### Biofilm formation assay

The measurement of biofilm formation of tested isolates was performed according to a standard protocol with little modification (Hashem et al. 2021). After isolation of the tested *Enterococcus* isolates, a pure colony of each isolate was inoculated in 10 ml Trypticase Soya broth (TSB) supplemented with 1% glucose at 37 °C overnight, and then 20 μL from the overnight bacterial culture was transferred to 96 well culture plate; to which 180 μL of TSB media + 1% glucose was added to adjust the OD with 10^8^ cells/mL at 600 nm. The plate was incubated at 37 for 24 h, after decantation of the content of the plate, the plate was gently washed twice with 300 μL phosphate buffer saline (PBS), and then the oven was used at 55 °C for 60 min for heat fixation (Stepanović et al. 2007).

After that, the plate was washed twice with sterile PBS to remove excess cells. This was followed by adding 150 μL of 1% crystal violet to each well for 15 min and then aspirating the stain, this was followed by washing the plate twice with sterile distilled water, and 150 μL of 95% ethanol was added to each well for 30 min to resolubilize the cell. The ELISA plate reader was used at 490 nm to determine the OD of each isolate. The absorbance value of each sample (ODS) was compared to the optical density cut-off (ODc) which was defined as three standard deviations above the mean OD of the negative control and the mean absorbance for each sample was calculated. Based on these comparisons, the samples were categorized into strongly forming biofilms (4 × ODc < ODs), moderately forming biofilms (2 × ODc < ODs ≤ 4 × ODc), and weakly forming biofilms (ODc < ODs ≤ 2 × ODc). Isolates with absorbance values equal to or lower than the sterility control were classified as non-biofilm producers. This result interpretation was done as previously reported (de Azevedo Ramos et al. 2023; Lin et al. 2023).

### Isolation and propagation of bacteriophage

Fifteen sewage samples (SS1-SS15) were collected from the diagnostic laboratories of Al Kasr Al-Einy University hospitals in Cairo, Egypt, an–n based on the high probability of the presence of the enterococci in them and, consequently, the presence of the bacteriophages with their host. From the sewage samples SS4, SS11, SS12, SS15, used for phage isolation, four phage lysates coded vB_EF_Enf1, vB_EF_Enf2, vB_EF_Enf3, and vB_EF_Enf4 were isolated, respectively. The Enterococcus phage vB_EF_Enf3 was isolated from the sewage sample SS12, identified, and deposited in the Culture Collection Ain Shams University (CCASU) (https://ccinfo.wdcm.org/collection/by_id/1186), under the code, Enterococcus phage vB_EF_Enf3_CCASU-2024-3. The isolation started first with samples' filtration with double filter paper in a 50 mL double strength TSB flask with traces of calcium carbonate and magnesium sulfate inoculated with 5 mL of bacterial culture in exponential phase, then 5 mL from filtrated sewage sample with a ratio 10:1:1 and incubated overnight at 28 °C at 160 rpm. After incubation, 10 mL of the co-culture was transferred in a sterile falcon tube and centrifuge at 4 °C at 6000 rpm for 35 min (Abd-Allah et al. 2022), the supernatant was filtered using a 0.22 nm (Agilent, CA, USA) syringe filter and this filtered lysate was examined for its lytic activity by plaque assay and spot test using double layer agar plate. The same aforementioned steps used in the isolation of bacteriophage were used in phage propagation, with the substitution of 5 mL from sewage with 5 mL from our filtrated lysate to confirm the increase in quantity and purity of the isolated phage after propagation. The plaque assay must be done for verification (Abd-Allah et al. 2022). For quantitative determination of phage titer, the following equation was used (Hallajzadeh et al. 2020; Abd-Allah et al. 2022).

Phage forming unit per ml (PFU/mL) = Number of plaques/(volume of purified phage used in each plate × dilution factor).

### Detection of the morphology of the isolated phage

High quantity and purity are very critical in this test. So, a propagated suspension of the phage lysate was prepared to obtain high phage titer to be examined using a transmission electron microscope (TEM), and this lysate was filtrated using a 0.22 mm (Agilent, CA, USA) syringe filter. To ensure purity, 50 μL from the purified lysate was transferred to sterile Eppendorf and was examined using TEM et al.-Azhar University, Cairo, Egypt as previously described (Raza et al. 2018; Mahmoud et al. 2021).

### Thermal and pH stability assay

For pH stability, the SM buffer was adjusted at different pH ranges from (1–12) using NaOH and HCL mixture and incubated at room temperature for 1 h, and phage lytic activity was examined by standard spot test (Mohammadi et al. 2023). For temperature stability, aliquots of the purified phage with fixed volume were placed in a water bath at temperatures 30, 35, 40, 45, 50, 55, 60, 65, 70, 75, and 80 °C for 1 h for each, and the lytic activity was determined by standard spot test (Abd-Allah et al. 2022; El-Atrees et al. 2022).

### Host range

A fixed volume (15 μL) from the isolated purified propagated phage lysate was tested against the collected *Enterococcus* isolates to examine its lytic activity and to determine its spectrum of activity using standard spot techniques as previously reported (Abd-Allah et al. 2022; El-Atrees et al. 2022).

### Phage whole genome characterization and annotation:

#### Genomic DNA extraction

The genomic DNA was extracted using the QIAamp® DNA mini kit (Qiagen, Hilden, Germany). The concentration, yield, and purity of the extracted DNA were determined for both quantity and quality, as specified in the kit's manual.

### Phage library, Oxford Nanopore sequencing, and ORF annotations

Genomic sequencing was performed using Oxford Nanopore sequencing (Wang et al. 2021) at HITS Solutions, Co, Cairo, Egypt (https://www.hitssolutions.com/). The genomic library was prepared using a rapid barcoding kit (SQK-RBK004; Oxford Science Park, OX4 4DQ, UK). The quality of the Fastq reads was assessed with FastQC, and reads that were too short or of low quality were removed using the NanoFilt tool (De Coster et al. 2018). Porechop_ABI was utilized to remove adapter sequences from the reads as previously described (Bonenfant et al. 2023) The filtered reads were then assembled de novo using Flye v2.9.3-b1797 (https://github.com/fenderglass/Flye) and polished with medaka v1.11.3 (https://github.com/nanoporetech/medaka). The final consensus sequence was structurally annotated using Prokka v1.14.5 and GeneMarkS v4.28 (Besemer 2001). The open reading frames (ORFs) were manually curated with FramePlot v4.0beta, using the following parameters: minimum ORF size of 40 codons; start codons ATG, GTG, TTG; incomplete ORFs excluded and the functional annotation was performed using BLAST® (Altschul et al. 1990). The final genomic sequence of the phage was submitted to the NCBI GenBank database under the accession PP747318 for Enterococcus phage_VB-EF_EnF3. The circular map of the phage genome was constructed using the BLAST Ring Image Generator (BRIG) tool v0.95 (Alikhan et al. 2011).

## Results

### Isolation and identification of enterococci:

Out of the collected bacterial isolates, 65 were identified to be enterococci based on Gram staining, and giving brown, black colonies on Bile Esculin Agar (BEA) medium (Fig. S1), negative catalase test, and positive 6.5% NaCl tolerance test. The frequency of enterococci from urine, blood, and pus specimens were n = 40 (61.53%), n = 15 (23.07%), and n = 10 (15.38%), respectively. The identification was confirmed by the VITEK 2 system, where 41.5% (n = 27) of the isolates were identified as *E. faecalis,* 50.7% (n = 33) as *E. faecium*, 4.6% (n = 3) as *E. avium,* and 3% (n = 2) as *E. durans.*

### Antibiotic susceptibility test:

**Fig. 1:**
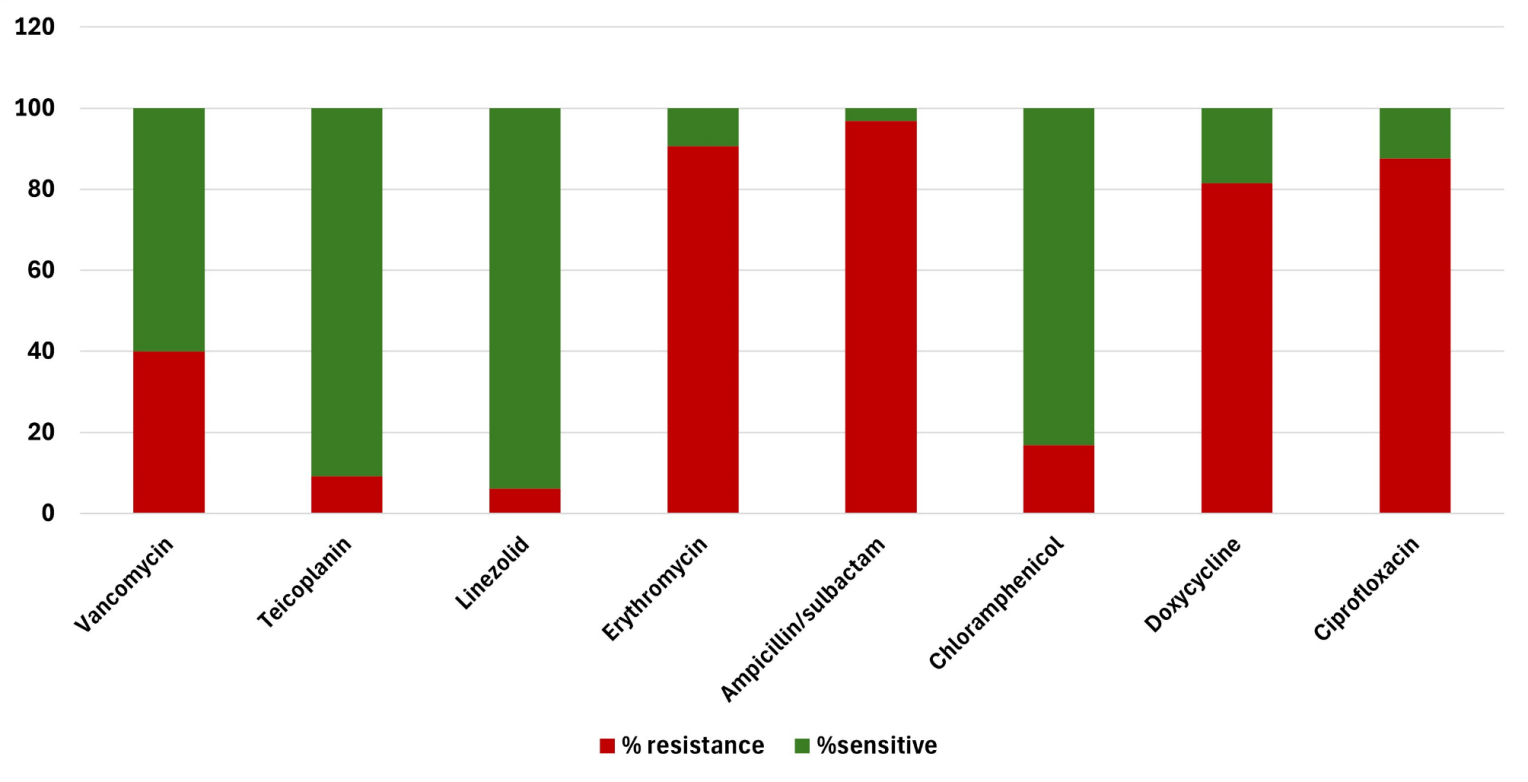
Results of the antibiotic susceptibility testing of the *Enterococcus* clinical isolates against eight antibiotics according to CLSI guidelines (n = 65)

Out of the collected *Enterococcus* isolates (n = 65), the highest resistance was observed against ampicillin/sulbactam, (96.9%; n = 63), followed by erythromycin (90.7%; n = 59), ciprofloxacin (87.6%; n = 57) and doxycycline (81.5%; n = 53). However, linezolid, teicoplanin, chloramphenicol, and vancomycin exhibited maximum sensitivity among the tested isolate with 6.15% (n = 4), 9.2%; (n = 6), 16.9%; (n = 11), and 40% (n = 26), respectively (Fig. 1; Table S1). As displayed in Table 1, a total of 87.69% (n = 57) of the tested isolates exhibited MDR phenotype**.** Results showed 26 (40%) out of tested 65 *Enterococcus* isolates were vancomycin-resistant enterococci (VRE) and 39 (60%) were vancomycin-sensitive enterococci (VSE) as delineated in Table 1.

**Table 1:** Kirby-Bayer antibiotic sensitivity testing of the collected *Enterococcus* clinical isolates

Isolate code	Isolate name	VAN	TEI	LZD	ERY	SAM	DOX	CMP	CIP	MDR phenotype
E1	*E. faecalis*	S	S	S	R	R	R	R	R	+
E2	*E. faecalis*	R	S	S	R	R	R	S	R	+
E3	*E. faecium*	R	S	S	R	R	R	S	R	+
E4	*E. faecium*	R	S	S	R	R	R	S	R	+
E5	*E. faecalis*	S	S	S	R	R	R	R	R	+
E6	*E. faecalis*	S	S	S	S	R	s	S	S	−
E7	*E. faecium*	S	S	S	S	R	s	S	S	−
E8	*E. faecium*	S	S	S	R	R	R	S	R	+
E9	*E. faecium*	S	S	S	R	S	I	S	R	−
E10	*E. faecium*	R	R	S	R	R	R	S	R	+
E11	*E. faecalis*	S	S	S	S	R	R	R	R	+
E12	*E. faecium*	S	S	S	R	R	R	S	R	+
E13	*E. faecium*	R	R	S	R	R	R	S	R	+
E14	*E. faecalis*	S	S	S	S	R	R	S	S	−
E15	*E. faecalis*	S	S	S	R	R	R	S	R	+
E16	*E. faecalis*	S	S	S	R	R	R	S	R	+
E17	*E. faecium*	R	S	S	R	R	R	S	R	+
E18	*E. faecium*	R	S	S	R	R	R	S	R	+
E19	*E. avium*	R	S	S	R	R	R	S	R	+
E20	*E. faecium*	R	S	S	R	R	S	I	R	+
E21	*E. faecalis*	R	S	S	R	R	R	S	R	+
E22	*E. faecium*	R	S	S	R	R	S	S	R	+
E23	*E. faecium*	R	S	S	R	R	S	S	R	+
E24	*E. faecalis*	S	S	S	I	R	S	I	I	−
E25	*E. faecium*	S	S	S	R	R	R	I	R	+
E26	*E. faecalis*	S	S	S	R	R	R	S	R	+
E27	*E. faecium*	S	S	S	R	R	R	R	R	+
E28	*E. faecalis*	S	S	S	R	R	R	S	R	+
E29	*E. durans*	S	S	S	R	R	R	I	I	+
E30	*E. faecium*	S	S	S	R	R	R	S	R	+
E31	*E. faecalis*	S	S	S	R	R	R	S	R	+
E32	*E. faecium*	S	S	S	R	R	R	S	R	+
E33	*E. faecalis*	S	S	S	R	R	R	S	R	+
E34	*E. faecium*	S	S	S	I	R	S	S	S	−
E35	*E. avium*	S	S	S	R	R	R	R	R	+
E36	*E. faecium*	S	S	S	R	R	S	S	S	−
E37	*E. faecium*	S	S	S	R	R	R	R	R	+
E38	*E. durans*	S	S	S	R	R	R	R	R	+
E39	*E. avium*	S	S	S	R	R	R	S	R	+
E40	*E. faecalis*	S	S	S	R	R	R	S	R	+
E41	*E. faecalis*	S	S	S	R	R	s	S	R	+
E42	*E. faecalis*	S	S	S	R	R	R	R	R	+
E43	*E. faecalis*	S	S	S	R	R	R	S	R	+
E44	*E. faecium*	S	S	S	R	R	R	S	R	+
E45	*E. faecium*	S	S	S	R	R	s	S	S	−
E46	*E. faecalis*	S	S	S	R	R	R	R	R	+
E47	*E. faecalis*	S	S	S	R	R	I	S	R	+
E48	*E. faecalis*	R	S	S	R	R	R	S	R	+
E49	*E. faecalis*	S	S	S	R	R	R	S	R	+
E50	*E. faecium*	R	R	S	R	R	R	S	R	+
E51	*E. faecalis*	R	S	S	R	R	R	S	R	+
E52	*E. faecium*	R	I	R	R	R	R	I	R	+
E53	*E. faecalis*	S	S	S	R	R	R	S	R	+
E54	*E. faecalis*	R	R	S	R	R	R	S	R	+
E55	*E. faecalis*	S	S	S	R	S	R	S	R	+
E56	*E. faecium*	R	S	S	R	R	R	I	R	+
E57	*E. faecium*	R	S	R	R	R	R	I	R	+
E58	*E. faecium*	R	R	R	R	R	R	R	R	+
E59	*E. faecium*	R	S	S	R	R	R	S	R	+
E60	*E. faecalis*	S	S	S	R	R	R	S	R	+
E61	*E. faecium*	R	S	S	R	R	R	S	R	+
E62	*E. faecium*	R	S	S	R	R	R	S	R	+
E63	*E. faecium*	R	S	S	R	R	R	I	R	+
E64	*E. faecium*	R	S	S	R	R	R	S	R	+
E65	*E. faecium*	R	R	R	R	R	R	R	R	+

*Van* vancomycin; *TEI* teicoplanin; *ERY* erythromycin; *AMC* amoxicillin-clavulanic acid; *DOX* doxycycline, *CMP* chloramphenicol; *CIP* ciprofloxacin; *MDR* multidrug-resistant; *S* sensitive; *R* resistant; *E*. *Enterococcus*

### Detection of biofilm formation and gelatinase production:

**Fig. 2:**
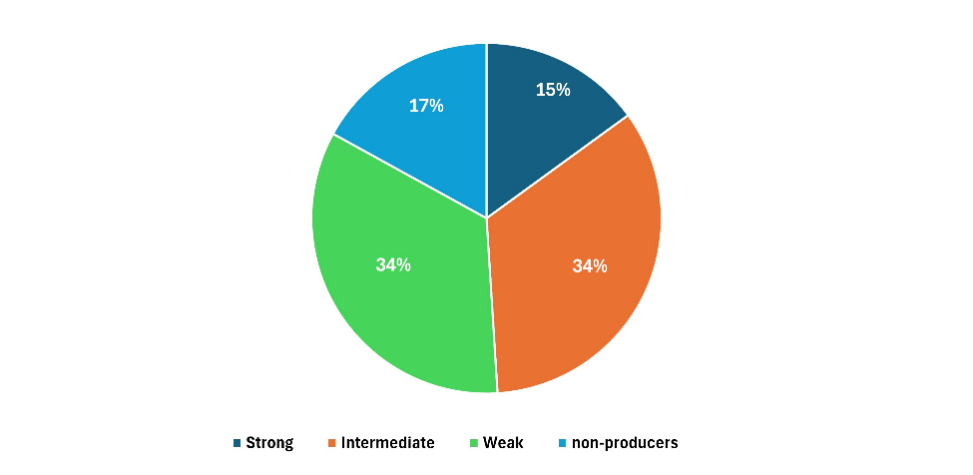
Distribution of biofilm formation of the recovered *Enterococcus* clinical isolates (n = 65)

As shown in Table 2, out of the 65 tested isolates; 25 (38.5%) isolates were gelatinase- and biofilm-producers, while out of the 39 gelatinase non-producers, 29 (44.6%) were biofilm producers. On the other hand, only one gelatinase-producing isolate was non-biofilm producer. Data were evaluated using the Chi-square test and a significant correlation was detected (*P value* = 0.02). According to the type of specimens, the highest biofilm-producing *Enterococcus* isolates were those recovered from urine followed by blood and pus specimens with a percentage of 62.9%, 20.37% and 16.66%, respectively (Table 2). The magnitude of the biofilm formation among the respective *Enterococcus* clinical isolates was distributed as follows: strong, 10 (15%), intermediate 22 (34%); weak 22 (34%) and non-biofilm producers 11 (17%) as depicted in Fig. 2 and the result of each isolate is displayed in Table S2.

**Table 2:** The number and percentage of gelatinase- and biofilm-producers clinical isolates of enterococci (n = 65)

	Number of biofilm-producers (%)		Total	*P* value
	No	Yes		
Number of gelatinase-producers (%)				
No	10 (15.4%)	29 (44.6%)	39 (60%)	0.022
Yes	1 (1.5%)	25 (38.5%)	26 (40%)	
Specimen type				
Blood	4 (36.3%)	11 (20.37%	15 (23%)	0.022
Urine	6 (54.5%)	34 (62.9%)	40 (61.5%)	
Pus	1 (9.09%)	9 (16.66%)	10 (15.3%)	
Total	11 (16.9%)	54 (83.1%)	65 (100%)

### Bacteriophage vB_EF_Enf3 recovery and characterization

**Fig. 3:**
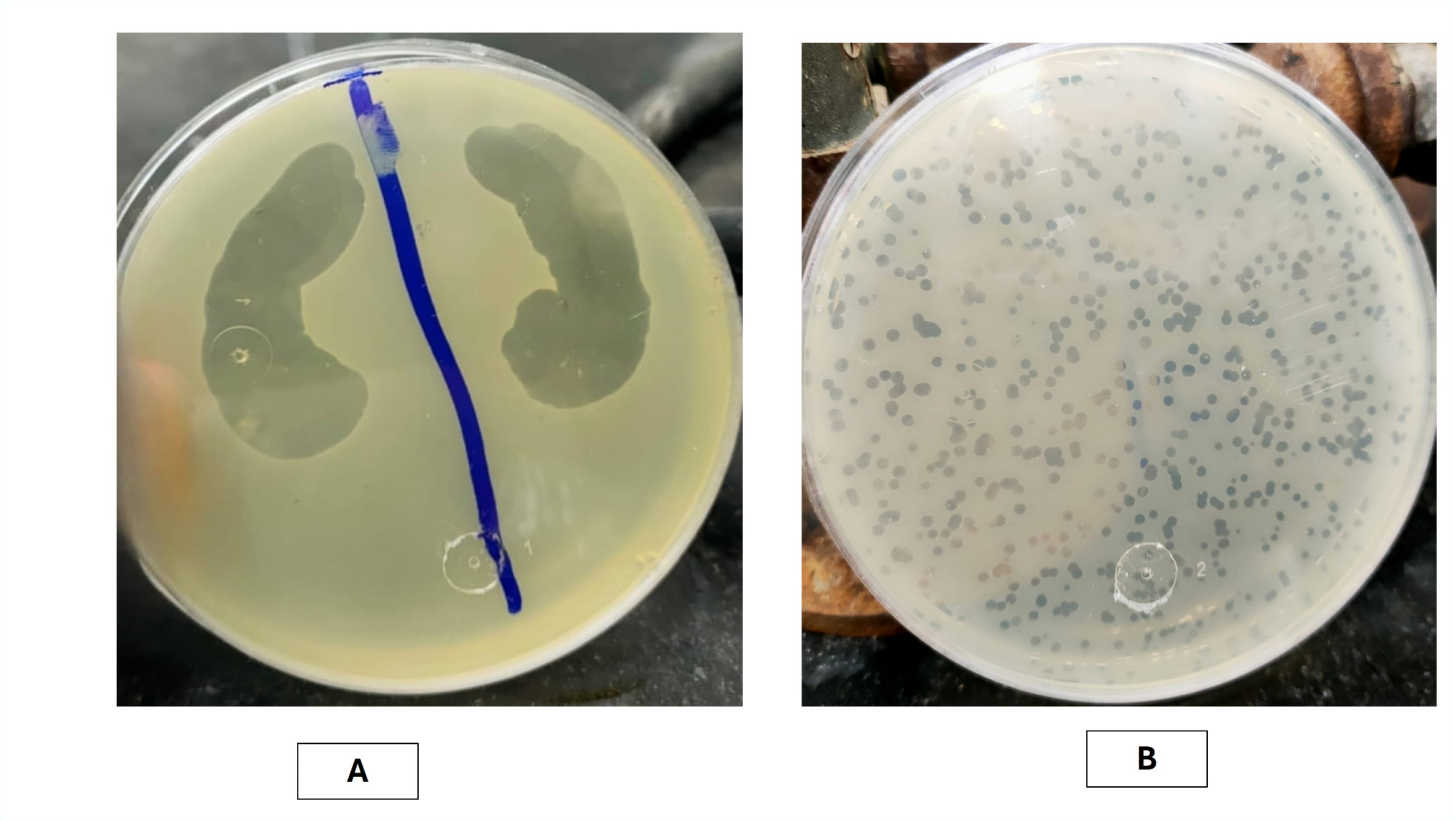
Enterococcus phage vB_EF_Enf3_CCASU-2024-3: **a** spot test appeared with clear transparent spot which prove its lytic activity, **b** plaque assay measured as PFU/mL

The four phage lysates coded vB_EF_Enf1, vB_EF_Enf2, vB_EF_Enf3, and vB_EF_Enf4 were isolated, and each gave lytic activity against *E. faecalis* isolate E1*, E. faecalis* isolate E2*, E. faecium* isolate E3 and *E. faecium* isolate E4, respectively (Table 1). However, vB_EF_Enf1, vB_EF_Enf2, and vB_EF_Enf4 lose their activity by repetitive testing, and we assume that they were lysogenic. On the contrary, the phage vB_EF_Enf3 gave a promising result in the spot test (Fig. 3a) and high initial titer (1.5 × 10^9^ PFU/mL) as calculated from plaque assay, and the formed plaques were clear, circular, and appeared regular in shape with a diameter between (1 and 5 mm) as shown in Fig. 3b.

### Transmission electron microscope demonstration:

**Fig. 4:**
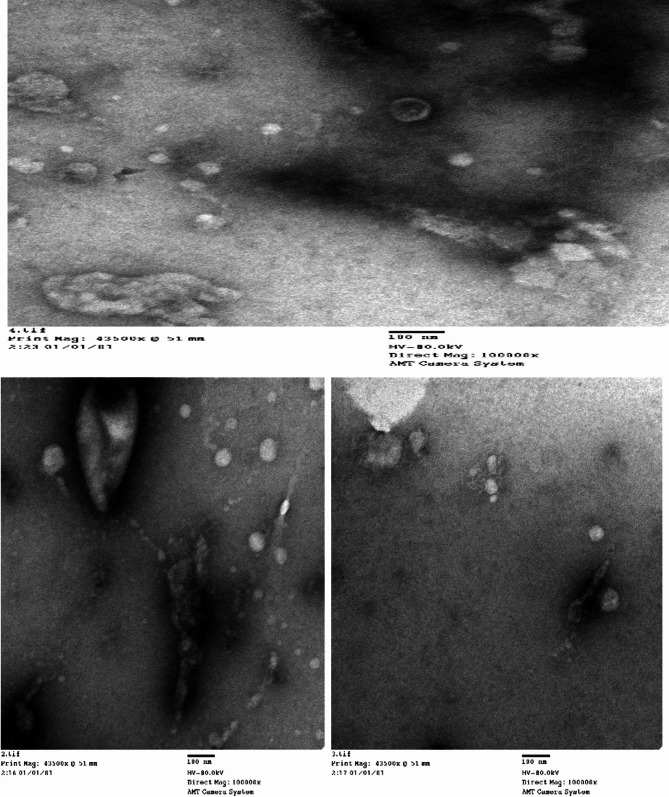
Transmission electron microscope (TEM) analysis of Enterococcus phage vB_EF_Enf3_CCASU-2024-3phage. The head is (100 nm) and tail length is (70 nm) with scale bar 100 nm

The results obtained using TEM analysis revealed that the Enterococcus phage vB_EF_Enf3_CCASU-2024-3 was a tailed bacteriophage indicating that it belongs to the order *Caudovirales* that is characterized by having a relatively large head (100 nm) and a long tail (70 nm) (Fig. 4).

### Thermal and pH stability of the Enterococcus phage vB_EF_Enf3_CCASU-2024-3

The Enterococcus phage vB_EF_Enf3_CCASU-2024-3 maintained its lytic activity at incubation temperature from 30 to 60 °C (Table S3) and at pH range from 3 to 8 (Table S4). However, at temperature range, 70–80 °C and at extreme acidic pH (1–2), and in alkaline pH (9–12), the infectivity of the phage was lost.

### Host range

The Enterococcus phage vB_EF_Enf3_CCASU-2024-3 showed lytic activity against three *E. faecium* clinical isolates coded E27, E34 and E36 as well as against four *E. faecalis* clinical isolates coded E11, E15, E47 and E60. The seven clinical *Enterococcus* isolates were deposited in the Culture Collection Ain Shams University (CCASU) (https://ccinfo.wdcm.org/collection/by_id/1186), under the codes, *E. faecium* CCASU*_*E27, *E. faecium* CCASU_E34, *E. faecium* CCASU*_*E36, *E. faecalis* CCASU_E11*, E. faecalis* CCASU_E15*, E. faecalis* CCASU_E47 and *E. faecalis* CCASU_E60.

### Whole genome sequencing of phage vB_EF_Enf3 and ORF prediction

**Fig. 5:**
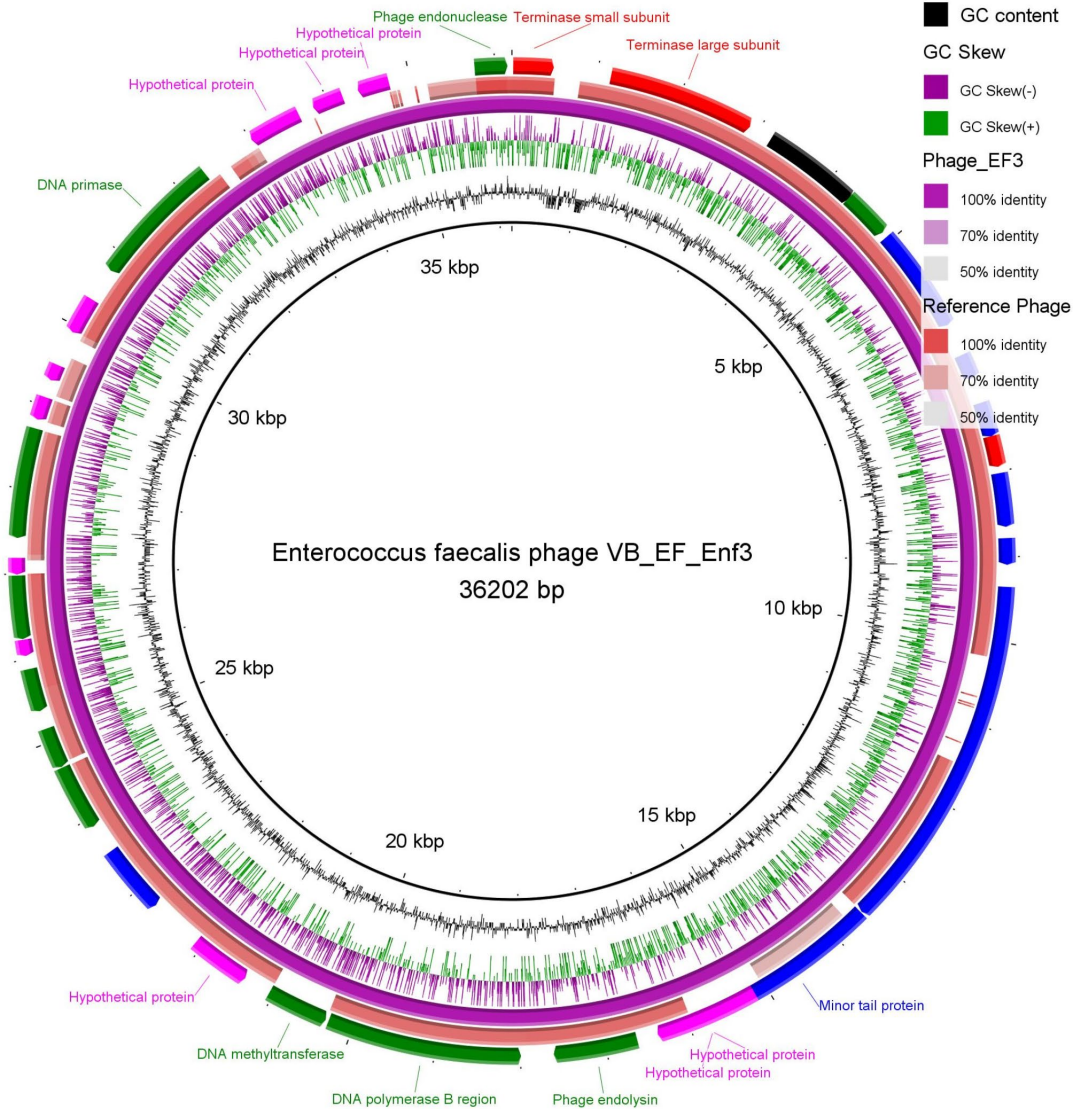
Circular genome map of Enterococcus phage vB_EF_Enf3_CCASU-2024-3 (NCBI GenBank Accession code, PP747318, size 36,202 bp, purple ring) and the reference phage (Enterococcus phage EFA1, complete genome; NCBI accession code, MT857001; size 40,454 bp, orange ring). The color coding of genes indicates the functional categories of putative proteins: Structural proteins (blue), terminase/terminator protein (red); non-structural proteins (green), portal proteins (black), hypothetical proteins (Fuchsia). The creation of the circular image was performed using the BLAST Ring Image Generator (BRIG) tool v0.95 (https://sourceforge.net/projects/brig/, accessed on 29 August 2024)

After assembling and annotating the genomic sequences of the phage EnF3, the genome was determined to be 36,202 bp in length with a G + C content of 34.4, containing 36 Open reading Frames (ORFs) as detailed in Table 3**.** These ORFs included 8 structural proteins, 11 non-structural proteins, 12 hypothetical proteins, 3 terminase proteins, and 2 portal proteins (Table S3). The genomic sequence has been submitted to the NCBI GenBank database under the accession number PP747318. BLASTn analysis provided the phage's taxonomical classification as follows: Viruses; (Duplodnaviria; Heunggongvirae; Uroviricota; Caudoviricetes; Efquatrovirus) Efquatrovirus SANTOR1, with a 92-query coverage and 94% identity. The circular genome map and the annotated ORF of phages EnF3 are delineated in Fig. 5**.**

**Table 3:** Feature annotations and open reading frame (ORF) analysis of Enterococcus phage vB_EF_Enf3_CCASU-2014-3

orf number	Feature ORF name	Strand	Interval range
1	Terminase small subunit [Enterococcus phage EFAP-1]	**+**	16…489
2	Terminase large subunit [Enterococcus phage vB_Efa_ZAT1]	**+**	1162…2889
3	portal protein [Enterococcus phage EfaCPT1]	**+**	3171…4322
4	Head maturation protease	**+**	4309…4872
5	capsid protein [Enterococcus phage SSMH01]	**+**	4942…6192
6	Head–tail adaptor Ad1 [Enterococcus phage IME-EF4]	**+**	6576…6872
7	Head–tail joining protein [Enterococcus phage EFRM31]	**+**	7176…7586
8	Tail terminator [Enterococcus phage EFRM31]	**+**	7583…7948
9	Major tail protein [Enterococcus phage phiSHEF2]	**+**	8024…8653
10	Head–tail adaptor [Enterococcus phage EFAP-1]	**+**	8785…9096
11	Tail tape measure [Enterococcus phage vB_EfaS_785CS]	**+**	9353…13585
12	Minor tail protein	**+**	13597…15654
13	Hypothetical protein	**+**	15624…15956
14	Hypothetical protein	**+**	16153…16380
15	Holin protein	**+**	16395…16631
16	Phage endolysin	**+**	16628…17614
17	DNA polymerase B region	**−**	Complement (17996…20287)
18	DNA methyltransferase	**−**	Complement (20323…21051)
19	Hypothetical protein	**−**	Complement (21085…21309)
20	Hypothetical protein	**−**	Complement (21379…22089)
21	Flagellar hook-associated protein	**−**	Complement (22724…23542)
22	putative ribonuclease	**−**	Complement (23894…24670)
23	Phage endonuclease	**−**	Complement (24681…25160)
24	Nucleotide kinase	**−**	Complement (25377…25883)
25	Hypothetical protein	**−**	Complement (26040…26231)
26	Putative DNA replication protein	**−**	Complement (26239…26982)
27	Hypothetical protein	**−**	Complement (26994…27188)
28	Helicase	**−**	Complement (27420…28715)
29	Hypothetical protein	**−**	Complement (28822…29097)
30	Hypothetical protein	**−**	Complement (29305…29499)
31	Hypothetical protein	**−**	Complement (29918…30361)
32	DNA primase	**−**	Complement (30762…32342)
33	Hypothetical protein	**−**	Complement (33012…33635)
34	Hypothetical protein	**−**	Complement (33824…34186)
35	Hypothetical protein	**−**	Complement (34380…34751)
36	Phage endonuclease	**+**	35767…36150

## Discussion

It has become crystal clear how dangerous and significant enterococci are due to their ability to evolve and adapt to harsh ecological conditions. This adaptability is largely due to their acquisition of resistance genes, which enable them to withstand antibiotics, and their production of virulence factors such as gelatinase and biofilm formation, both of which contribute to the pathogenesis of enterococcus infection (Arshadi et al. 2018). Biofilm plays a pivotal role in bacterial survival in host tissue and immune modulation, while gelatinase, a zinc-containing metalloprotease, aids in host tissue invasion and bacterial attachment and colonization by hydrolyzing gelatin, collagen, and other peptides (Fahmy et al. 2021; Shahi et al. 2020).

Since enterococci are controversial, they have attracted the attention of many researchers who aim to evaluate their virulence determinants and the correlations between them. There is also a focus on finding alternatives to traditional treatment solutions, especially for diseases caused by MDR enterococci (Saffari et al. 2017; Al-Zubidi et al. 2019). In our study, sixty-five enterococcus clinical isolates were collected. The percentage of *E. faecalis* and *E. faecium* were 41.5% and 50%, respectively. Although *E. faecalis* is generally more prevalent, there has been a notable increase in the prevalence of *E. faecium*, especially in resistant strains of enterococci, over recent years (Saba Copur et al. 2016). In the distribution of *Enterococcus* species, *E. faecalis* and *E. faecium* are the most frequently encountered in human diseases (Tkachev et al. 2022).

Based on the result, the antibiotics that showed the lowest activities on the collected isolates were erythromycin, ampicillin, gentamicin, and ciprofloxacin. This finding is consistent with the results of Shahi et al. (Shahi et al. 2020). This resistance can be explained by the excessive use of unprescribed antibiotics and the disregard for using susceptibility test results (Arshadi et al. 2018). We also discovered that linezolid is considered the last antibiotic option for VRE isolates; however, unfortunately, four isolates were identified as linezolid-resistant enterococci. According to our results, we deduce that there is a significant correlation between gelatinase production and biofilm formation, consistent with the findings of Saffari et al. (Saffari et al. 2017). Fahmy et al. explained that biofilm-positive isolates with negative gelatinase production could be due to the silent gelE gene in these isolates (Fahmy et al. 2021). This gene aids in biofilm production but is not phenotypically expressed, as its expression depends on other genes, which is congruent with Hashem et al. (Hashem et al. 2021). In contrast, Shahi et al. described the biofilm formation process as a multifactorial process dependent on environmental conditions rather than solely on virulence determinants such as gelE and gelatinase production (Shahi et al. 2020). It is also obvious that the origin of the specimen affects the biofilm production ability of the isolates, with a higher prevalence of biofilm production observed in urine specimens compared to other types. A logical explanation for this is that biofilm helps the bacteria to attach, colonize, and protect themselves from being washed out by urine (Shahi et al. 2020). This finding aligns with the results of Hashem et al. (Hashem et al. 2021). There is no doubt that we are at the beginning of a real catastrophe due to the exacerbation of the diseases caused by MDR bacteria and the lack of effective antibiotic solutions (Mabrouk et al. 2020; Abdelaziz et al. 2021). However, there is a glimmer of hope in addressing this predicament, represented by bacteriophages. These natural predators of bacteria are considered the most ubiquitous and genetically diverse entities on Earth (Wang et al. 2014; El-Atrees et al. 2022). In the present study, isolation and characterization of the bacteriophage vB_EF_Enf3 was undertaken from sewage water, as noted in previous studies (Kabwe et al. 2021; El-Atrees et al. 2022). These studies have attributed the presence of bacteriophages in sewage to its high content of bacteria and other organic matter. To be considered for therapeutic use, phages must meet certain characteristics, including host range, stability, and lytic activity. Bacteriophages have specific host ranges based on the genus, species, and strains they can infect. This is very crucial for phage therapy. Limiting a phage's host range to a single species keeps it from attacking bacteria other than the disease-causing one, maintaining the host's microbiome and avoiding harmful consequences for patients. For species, a phage with a limited host range is preferred. However, a phage that can lyse the majority of strains in this species is economically beneficial. This demonstrates that it is suitable for empirical treatment, much like broad-spectrum antibiotics (Hyman 2019; Abd-Allah et al. 2023). The merit of using phage therapy as one shot is associated with its ability to multiply at the site of infection where its selective bacterial host is copious also phages devour their specific bacterial species without any prejudice on the commensal microbiome (Khalifa et al. 2016).

From the appearance of the plaques, we can deduce that our phage is a virulent phage, characterized by clear, transparent plaques, according to the findings of Abd-Allah et al. (Abd-Allah et al. 2022) and Park et al. (Park et al. 2020). The VB_EF_Enf3 phage appears to be stable across a broad temperature range (30–60 °C) and pH tolerance range (3–9). These results somewhat align with those of the other Enterococcus phages, such as vB-_EfaS_PHB08 (Yang et al. 2020) and Enterococcus phage vB_EfaS_HEf13 (Lee et al. 2019). Upon host range assay, the bacteriophage was strongly effective against three *E. faecium* and four *E. faecalis* clinical isolates indicating a broad spectrum of activity. Regarding thermal, and pH stability, as well as host range results, the vB_EF_Enf3 bacteriophage could be prognosticated as an excellent candidate for the pharmaceutical form used in therapy. Phage particles are initially classified based on their morphology. VB_EF_Enf3 is distinguished by a long tail (70 nm) and a large head (100 nm). The isolated phages VB_EF_Enf3 may belong to the order *Caudoviricetes* based on their features, which align with the (ICTV)-ninth report recommendations (King et al. 2012). Only genomic characterization can precisely identify isolated phages, not their morphological characteristics. To verify and categorize the recovered phage, we used genomic sequencing, which is considered the gold standard (Youssef et al. 2024). The genotypic examination of phage VB_EF_Enf3 categorized it as a virus (*Duplodnaviria; Heunggongvirae; Uroviricota; Caudoviricetes; Efquatrovirus*). Based on such promising results, the Enterococcus phage vB_EF_Enf3_CCASU-2024-3 is considered a very promising candidate for pharmaceutical formulation as topical or oral preparations, and preclinical and clinical evaluation for combating infection *Enterococcus* infections in humans. In conclusion, linezolid remains the final antibiotic option against VRE clinical isolates. A novel lytic bacteriophage vB_EF_Enf3, belongs to the class *Caudoviricetes,* within the genus *Efquatrovirus* isolated from sewage water. This phage was evaluated in vitro*,* where it demonstrated strong lytic broad-spectrum activity against both *E. faecium and E. faecalis.* It exhibited good stability under extreme conditions, including variations in temperature and pH range. These characteristics make it a promising and appropriate candidate for in vivo testing and pharmaceutical formulation for its potential use in the management of *Enterococcus* infection in humans.

## Electronic supplementary material

Below is the link to the electronic supplementary material.

Supplementary Material 1.

## Data Availability

All data generated or analyzed during this study are included in this published article. The genomic sequence of Enterococcus phage vB_EF_Enf3_CCASU-2024-3 has been deposited in the NCBI GenBank database under the accession number PP747318 (https://www.ncbi.nlm.nih.gov/nuccore/PP747318).

## Abbreviations

BHI: Brain heart infusion
ESP: Enterococcal surface protein
CylA: Cytolysin
GelE: Gelatinase
UTIs: Urinary tract infections
FDA: Food and drug administration
MIC: Minimum inhibitory concentration
PFU: Plaque forming unit
PBS: Phosphate buffer saline
PVDF: Polyvinylidene difluoride
TEM: Transmission electron microscopy
TSB: Tryptic soy broth
VRE: Vancomycin-resistant enterococci
LRE: Linezolid-resistant enterococci
