# Supplementary material for: A novel thermostable lytic phage vB_EF_Enf3_CCASU-2024-3 against clinical Enterococcus faecium and Enterococcus faecalis
Source: AMB Express. 2025 Apr 26;15:65. doi: 10.1186/s13568-025-01871-z (PMC12033158; doi:10.1186/s13568-025-01871-z)
Supplement: Supplementary file 1 — Supplementary Material 1. [file 13568_2025_1871_MOESM1_ESM.docx]

**Table S1.** Antibiotic susceptibility test results of *Enterococcus* sp isolates (n=65).

| **Antibiotic** | **Number of resistant isolates** | **Percentage %** |
| --- | --- | --- |
| Vancomycin | 26 | 40 |
| Teicoplanin | 6 | 9.23 |
| Linezolid | 4 | 6.15 |
| Erythromycin | 59 | 90.7 |
| Ampicillin/sulbactam | 63 | 96.9 |
| Chloramphenicol | 11 | 16.92 |
| Doxycycline | 53 | 81.53 |
| Ciprofloxacin | 57 | 87.69 |

**Table S2**. Biofilm formation test results (n=65)

| **Isolate code** | **No biofilm** | **Week biofilm** | **Intermediate biofilm** | **Strong biofilm** |
| --- | --- | --- | --- | --- |
| E1 |  |  | + |  |
| E2 |  |  |  | + |
| E3 |  | + |  |  |
| E4 |  | + |  |  |
| E5 |  |  |  | + |
| E6 |  |  | + |  |
| E7 |  |  | + |  |
| E8 |  | + |  |  |
| E9 |  | + |  |  |
| E10 |  |  | + |  |
| E11 |  |  |  | + |
| E12 |  |  | + |  |
| E13 |  |  | + |  |
| E14 |  |  |  | + |
| E15 |  | + |  |  |
| E16 |  |  | + |  |
| E17 |  |  | + |  |
| E18 |  |  | + |  |
| E19 |  |  | + |  |
| E20 |  |  |  | + |
| E21 |  |  | + |  |
| E22 |  | + |  |  |
| E23 | + |  |  |  |
| E24 |  |  |  | + |
| E25 |  |  | + |  |
| E26 |  |  |  | + |
| E27 |  | + |  |  |
| E28 |  | + |  |  |
| E29 |  |  | + |  |
| E30 |  | + |  |  |
| E31 |  | + |  |  |
| E32 |  |  | + |  |
| E33 |  | + |  |  |
| E34 |  |  |  | + |
| E35 |  | + |  |  |
| E36 |  |  | + |  |
| E37 |  | + |  |  |
| E38 |  |  | + |  |
| E39 |  | + |  |  |
| E40 |  |  | + |  |
| E41 |  |  | + |  |
| E42 |  |  |  | + |
| E43 |  |  | + |  |
| E44 |  |  | + |  |
| E45 |  |  | + |  |
| E46 |  |  |  | + |
| E47 |  |  | + |  |
| E48 | + |  |  |  |
| E49 | + |  |  |  |
| E50 | + |  |  |  |
| E51 |  | + |  |  |
| E52 |  | + |  |  |
| E53 | + |  |  |  |
| E54 | + |  |  |  |
| E55 |  | + |  |  |
| E56 |  | + |  |  |
| E57 | + |  |  |  |
| E58 | + |  |  |  |
| E59 | + |  |  |  |
| E60 |  | + |  |  |
| E61 |  | + |  |  |
| E62 |  | + |  |  |
| E63 | + |  |  |  |
| E64 | + |  |  |  |
| E65 |  | + |  |  |

**Table S3**. Thermal stability test of *Enterococcus* phage vB_EF_Enf3_CCASU-2024-3

| Temperature | Spot test |
| --- | --- |
| 30°C | + |
| 40°C | + |
| 50°C | + |
| 60°C | + |
| 70°C | - |
| 80°C | - |

**Table S4.** pH stability test for *Enterococcus* phage vB_EF_Enf3_CCASU-2024-3

| **pH value** | **Spot test** |
| --- | --- |
| 1 | - |
| 2 | - |
| 3 | + |
| 4 | + |
| 5 | + |
| 6 | + |
| 7 | + |
| 8 | + |
| 9 | - |
| 10 | - |
| 11 | - |
| 12 | - |


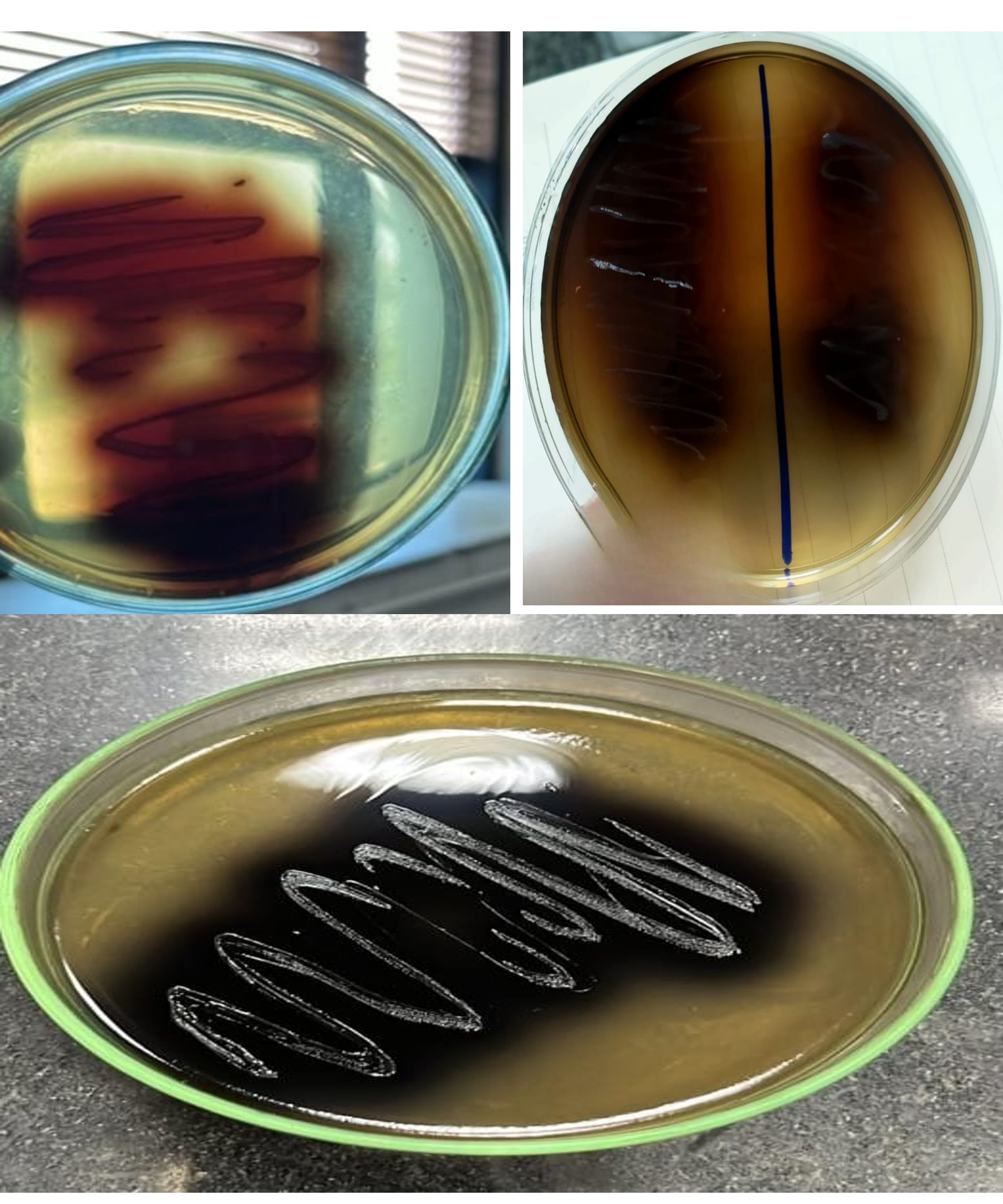


**Fig. S1**. Phenotypic growth of *Enterococcus* sp. showing brown, black colonies on Bile Esculin Agar (BEA) plates
